# A novel exopolysaccharide-producing bacterium, *Pseudescherichia liriopis* sp. nov. isolated from *Liriope platyphylla*, enhances the growth of *Daucus carota* subsp. *sativus* under drought and salinity stress

**DOI:** 10.3389/fpls.2024.1417639

**Published:** 2024-07-16

**Authors:** Inhyup Kim, Haejin Woo, Geeta Chhetri, Sunho Park, Taegun Seo

**Affiliations:** Department of Life Science, Dongguk University-Seoul, Goyang, Republic of Korea

**Keywords:** PGPB, plant-microbe interaction, stress tolerance in plants, exopolysaccharides, phylogenetic analysis

## Abstract

Biological and abiotic stresses in plant growth are associated with reduced crop yields. Therefore, improving plant stress resistance can be a crucial strategy to improve crop production. To overcome these problems, plant growth-promoting bacteria are emphasized as one of the alternative tools for sustainable agriculture. This study found a novel strain (L3^T^) of a plant growth-promoting bacterium in fermented *Liriope platyphylla* fruit. Strain L3^T^ showed the ability to promote plant growth. The L3^T^ strain promoted plant growth of *D. carota* subsp. *sativus*, increasing the length (increase rate compared to the control group, 36.98%), diameter (47.06%), and weight of carrots (81.5%), ultimately increasing the edible area. In addition, we confirmed that plant growth was improved even in situations that inhibited plant growth, such as salinity and drought stress. Strain L3^T^ performed indole production, siderophore production, phosphate solubilization, and nitrogen fixation, all characteristics of a strain that promotes plant growth. Genome analysis revealed genes involved in the growth promotion effects of strain L3^T^. Additionally, the properties of exopolysaccharides were identified and characterized using FTIR, TGA, and UHPLC. Our results demonstrated that L3 isolated from fermented *L*. *platyphylla* fruit can be used to simultaneously alleviate drought and NaCl stress.

## Introduction

In recent years, many research teams have shown increasing interest in exploring the potential of microorganisms to continuously improve agricultural productivity (Mustapha et al., 2020; Yan et al., 2020; Thakur et al., 2023). Microorganisms play an important role in various ecological processes, such as promoting plant growth and nutrient cycling. Microorganisms like plant growth-promoting bacteria (PGPB) can positively affect plant growth and development. Among PGPB, the L3^T^ strain we discovered was isolated from fermented *Liriope platyphylla* fruit. This is claimed to be a new bacterial strain with remarkable properties that have the potential to benefit both plant and human health. The plant growth-promoting (PGP) strain ability was demonstrated by fermenting *L*. *platyphylla* fruits near Dongguk University in the Republic of Korea and then inoculating the isolated strain L3^T^ into carrot plants. In addition, this paper is a continuation of research that isolated a novel species from *L*. *platyphylla* (Kim et al., 2020b, 2023a).

*L. platyphylla* is used as a traditional medicine for cough and lung inflammation diseases in some Asian countries, including the Republic of Korea (Hur et al., 2004). As a result of recent research, anti-obesity, anti-inflammatory, and the potential for estrogenic, antiplatelet, and antiviral effects were discovered in *L. platyphylla* (Tsai et al., 2013; Huang et al., 2014; Kim et al., 2016; Le et al., 2021). The *L. platyphylla* fruit, which is relatively less studied than the root, contains anthocyanins, has excellent antioxidant potential, and has been shown to inhibit collagenase (Lee and Choung, 2011; Truong et al., 2023). Collagen, which primarily constitutes the dermal layer of the skin, is broken down by an enzyme called collagenase. Therefore, inhibiting collagenase activity is a promising strategy to prevent skin aging, as it plays a crucial role in maintaining skin elasticity and strength (Ohtsuki et al., 2008; Morikawa et al., 2020). Some bacteria are known to produce collagenase. These microorganisms provide nutrients by breaking down collagen-like proteins around plant roots or can influence the regulation of the plant’s immune response (Duarte et al., 2016; Bhagwat et al., 2018; Pequeno et al., 2019). Strain L3^T^, isolated from the fruit of *L*. *platyphylla*, has the ability to promote plant growth and is a producer of novel exopolysaccharides (EPS).

EPS are high-molecular-weight natural polymers produced by microorganisms, including bacteria, fungi, and blue-green algae (Angelin and Kavitha, 2020). Some microbial-derived EPS have several physiological functions attributed to their antiviral, anti-inflammatory, and antioxidant activities (Wu et al., 2021; Kim et al., 2022). In addition, EPS production is less affected by seasons than polysaccharides made from animals or plants is easy to handle and manage, and has industrial advantages and potential for various applications in medicine, food, and agriculture (Daba et al., 2021). In particular, EPS produced by PGPB greatly help promote plant growth and play a role in protecting plants from abiotic stress (Liu et al., 2017). EPS produced by plant-derived bacteria protects plants from abiotic stresses caused by adverse conditions. EPS from PGP bacteria alleviate NaCl stress by inhibiting Na⁺ uptake in plant roots and preventing translocation to leaves (Bhat et al., 2020). Moreover, bacterial EPS production is a crucial mechanism that aids survival under microenvironmental changes (Roberson and Firestone, 1992). EPS provide high moisturizing power, preventing both plants and bacterial cells from drying out, thereby enhancing their viability (Zhang et al., 2022). To withstand soil strategies to maintain high water content. This process helps sustain plant growth and prevents root desiccation (Naseem et al., 2018). Additionally, EPS not only protect plants from drought stress but also facilitate bacterial attachment to plant root (Skorupska et al., 2006; Bhagat et al., 2021).

In this study, *Pseudescherichia liriopis* sp. nov., isolated from fermented *L*. *platyphylla* fruit, demonstrates PGP properties. Additionally, its PGP ability was tested under conditions of salinity and drought overlapping stress, showing efficacy even under simultaneous drought and NaCl stress. This study provides insight into the abiotic stress tolerance of strain L3, suggesting that it can improve crop productivity and be considered a solution for eco-friendly and sustainable agriculture in response to increasing soil drought and salinization (Abdelaal et al., 2021; Hassani et al., 2021).

## Materials and methods

### Isolation of strain from fermented *L. platyphylla* fruit

A bunch of fresh *L. platyphylla* fruit was collected from a flowerbed located in Goyang, Gyeonggi, Republic of Korea (37°40′40.9′′ N, 126 °48′24.9′′ E). The collected 10 g of fruit and 10 g of glucose were added to a 50-mL round tube, sealed, and fermented at 25°C for 1 month. Subsequently, a standard dilution method was performed, in which 0.1 mL of the previously fermented fermentation broth was added to 0.9 mL of 0.85% sterile saline and repeated. Aliquots of 0.1 mL of the sample suspension were spread on *Lactobacilli* MRS agar (BD Difco, Franklin Lakes, NJ, USA) plates and incubated at 25°C for 3 days. Colonies were then picked and purified by streaking 3 times under the conditions mentioned above. Selected strains were stored in 25% glycerol (w/v) at −80°C.

### Indole-3-acetic acid

Auxin production was determined using a colorimetric method (Sachdev et al., 2009). After inoculating the strain into R2A medium supplemented with 0%, 0.05%, 0.1%, 0.2%, and 0.3% _L_-tryptophan, it was cultured for 48h at 25°C and 160 rpm with shaking and then centrifuged to collect the supernatant. Equal volumes of Salkowski’s reagent (9.8 mL of 35% perchloric acid and 200 μL of 0.5 M FeCl_3_) were added to the supernatant, mixed, and left in the dark for 30 min. Absorbance at 530 nm was measured using a spectrophotometer (Multiskan GO; Thermo Fisher Scientific, Waltham, MA, USA). The indole-3-acetic acid (IAA) concentration values of the strains were determined by substituting them into the IAA standard curve (5 µg/mL, 10 µg/mL, 20 µg/mL, 50 µg/mL, and 100 µg/mL).

### Selection of PGPB candidates

IAA-producing strains were evaluated for PGP properties, such as phosphate solubilization, siderophore production, and nitrogen fixation. To check phosphate solubilization, PVK (Pikovskaya, 1948) was used, and the composition of the medium is as follows: FeSO_4_·7H_2_O (0.001%), yeast extract (0.05%), dextrose (1%), (NH_4_)_2_SO_4_ (0.05%), Ca_3_(PO_4_)_2_ (0.5%), KCl (0.02%), MgSO_4_·7H_2_O (0.0%), agar (1.5%), and MnSO_4_·7H_2_O (0.001%). Cultures were incubated at 25°C for up to 7 days to develop a clear zone around colonies grown in PVK medium. Siderophore production was confirmed using CAS agar (Schwyn and Neilands, 1987). Briefly, qualitative tests for siderophore production were performed: strain cultures were plated on plates supplemented with 10% CAS and incubated at 25°C for 1 week. An orange halo around a strain colony indicates a positive result. The nitrogen fixation capacity of the strains was studied on Jensen’s medium, and the composition is as follows: agar (1.5%), Na_2_MoO_4_·2H_2_O (0.2%), FeSO_4_·7H_2_O (0.01%), NaCl (0.05%), MgSO_4_·7H_2_O (0.05%), K_2_HPO_4_ (0.1%), sucrose (2.0%), and CaCO_3_ (0.2%). Briefly, the strain culture was plated on Jensen’s medium and cultured at 25°C for 7 days. Visual confirmation of colony growth on Jensen’s medium indicates positive nitrogen fixation.

### Strains for NaCl and drought tolerance

The method for selecting NaCl tolerance strains followed the protocol described by Kim et al (Kim et al., 2020a). NaCl (from 0% to 10% at 1% intervals; w/v) and was measured at various concentrations. In summary, various concentrations of NaCl were added to 10 mL of R2A liquid medium, then sterilized, and 200 μL was transferred to a 96-well plate. Next, 2 μL of the strain culture cultured for 24h was inoculated and incubated at 25°C for 48h and measured at 600 nm using a UV-vis spectrophotometer. R2A broth was used as a negative control at each concentration supplemented with NaCl without strain injection. To assess drought resistance, R2A medium was supplemented with PEG 6000 (Steinheim, Germany) at different concentrations (from 0% to 25% at 5% intervals; w/v), and 2 μL of each of strain L3^T^ that had been incubated for 24h was added, followed by incubation at 25°C for 48h. The growth of the strains was compared to the negative control by measurement at 600 nm using a UV-vis spectrophotometer.

### Identification and growth condition of the isolate

Genomic DNA was extracted from strains using the TaKaRa MiniBEST Bacteria Genomic DNA Extraction Kit Ver. 3.0 (Takara Bio, Kusatsu, Japan). The 16S rRNA gene sequence of *Pseudescherichia* sp. L3^T^ was amplified using the universal bacterial primer sets 27F, 518F, 805R, and 1492R (Weisburg et al., 1991). The sequence was further verified by screening the 16S rRNA gene from the genome using ContEst16S, and the 16S rRNA gene sequence obtained from the genome yielded the same results as before (Lee et al., 2017). It was also used to assess the contamination of the genome sequence. Multiple sequences were aligned using MEGA 11 software and analyzed using Cluster X (Tamura et al., 2021). Phylogenetic trees were reconstructed based on neighbor-joining (NJ), maximum-likelihood (ML), and maximum-parsimony (MP) algorithms. The NJ and ML algorithms were implemented using the Kimura two-parameter model (Saitou and Nei, 1987). A min-mini heuristic was applied to the MP to compare with the NJ and ML phylogenetic trees (Fitch, 1971) Phylogenetic tree topologies were evaluated via bootstrap analysis based on 1000 replications (Felsenstein, 1985). The reference strain *P. vulneris* JCM 1688^T^ was obtained from the Japan Collection of Microorganisms (JCM, Tsukuba, Japan).

### Taxonomic analysis

The growth of the novel strain in different media, temperatures, NaCl concentrations, and pH levels followed the protocol (Kim et al., 2023b). The hydrolysis of Tween 80, DNA, casein, chitin, and carboxymethylcellulose, as well as Gram reaction, motility, oxidase, and catalase tests, were performed (Chhetri et al., 2021). Cells cultured on R2A agar at 25°C for 2 days were negatively stained using 3% uranyl acetate, and the morphology of the L3^T^ strain was observed using a transmission electron microscope (Libra 120; Zeiss, Oberkochen, Germany). Biochemical and enzymatic tests were performed using the API 20NE kit according to the manufacturer’s instructions (bioMérieux, NC, USA). To investigate the oxygen requirement of the strains, the oxygen in the anaerobic chamber was removed using oxygen absorption strips (Mitsubishi Gas Chemical, Tokyo, Japan) and monitored continuously.

### Whole genome sequencing and annotation

Genomic DNA of *Pseudescherichia* sp. L3^T^ was extracted using the TaKaRa MiniBEST Bacterial Genomic DNA Extraction Kit Ver. 3.0 (Takara Bio, CA, USA) following the manufacturer’s protocol. The concentration and quality of the genomic DNA were checked using a NanoDrop 2000 Spectrophotometer (Thermo Fisher Scientific, MA, USA). Genome sequencing of strain L3^T^ libraries was performed using the (Illumina, CA, USA) HiSeq X platform and assembled using the SPAdes ver. 3.15 *de-novo* assembler (Bankevich et al., 2012). The genome contamination and completeness of the novel strain L3^T^ were analyzed using the bioinformatics tool CheckM (https://ecogenomics.github.io/CheckM) (Parks et al., 2015). The genome of *Pseudescherichia* sp. L3^T^ was annotated using the NCBI Prokaryote Genome Automatic Annotation Pipeline (ncbi.nlm.nih.gov/genome/annotation_pork). The draft genome was analyzed using Prokaryotic Genome Annotation (Prokka) v1.14.6 (Seemann, 2014). The ANI of the novel strain and its phylogenetically close relatives was calculated using EzBioCloud’s e-service and KBase wrapper (Goris et al., 2007). The genomes of strain L3^T^ and the reference strain, genes involved in secondary metabolism were predicted using antiSMASH 6.0 (Blin et al., 2019).

### Plant inoculation experiment of strain L3^T^

The plant growth promotion study was conducted for 2 months, from August to October 2022. A flower pot with a diameter of 18 cm and a height of 15 cm was used in the experiment. Strain inoculation was divided into two groups: non-inoculated (control) and inoculated with strain L3^T^. Seeds were used after sterilization with minor modifications, as described by Chhetri et al (Chhetri et al., 2022). Briefly, seeds were first defatted with distilled water and then disinfected with 70% ethanol. Then, 0.5 mL of Tween 20 was added to 50 mL of 20% Clorox (v/v) and sterilized by shaking for 20 min. Cells of the strain L3^T^ were grown by shaking and culturing at 30°C and 160 rpm for 24h. The cells were collected by centrifugation at 8,000 × *g* for 5 min and washed with sterile distilled water to maintain an _OD_600 close to 0.7. Strains were inoculated by spraying 25 mL of cell suspension (or 25 mL of sterilized R2A medium as a control) into the soil around the plants once every 2 weeks.

### Inoculation under drought and salt stress

Carrot seeds were surface-disinfected with 70% ethanol for 3 min, dried, shaken with 20% Clorox (0.5 mL of Tween 20 added) for 20 min, and then washed five times with sterile distilled water. The seeds were soaked for 30 min under conditions i to viii, respectively, and then sown. Starting one week later, each group of plants was inoculated by spraying 25 mL of each of conditions i–viii into the soil around the plant roots once a week as follows: (i) 0.514 M NaCl; (ii) 0.514 M NaCl + L3^T^; (iii) 5% PEG; (iv) 5% PEG + L3^T^; (v) 10% PEG; (vi) 10% PEG + L3^T^; (vii) 0.514 M NaCl, and 5% PEG; (viii) 0.514 M NaCl, 5% PEG, and L3^T^. Strain L3^T^ used for plant inoculation was cultured for 48h, and then cells were collected by centrifugation at 8,000 x g for 5 min and washed with sterile distilled water to maintain the OD600 close to 0.7.

### Exopolysaccharide extraction and purification

To extract the EPS of strain L3^T^, the strain was first cultured in R2A medium for 24h. Then, 10 mL (1% of 1 L) of the culture was inoculated into 1 L of R2A supplemented with 1% glucose and cultured for 76h. The supernatant was obtained by centrifugation (8,000 × *g*, 15 min) and then centrifuged again to obtain a cell-free supernatant. Next, 14% trichloroacetic acid was added to the supernatant and shaken at 90 rpm for 30 min at 25°C. Afterward, centrifugation was performed again under the same conditions to remove denatured proteins, and this was repeated twice. Cold absolute ethanol stored at −20°C was added in an amount three times the volume of the upper layer, and the mixture was allowed to precipitate overnight in a 4°C refrigerator. The process was repeated twice. The precipitated EPS was separated, placed in a 50 mL round tube, and centrifuged (10,000 × *g*, 10 min) to completely remove ethanol. The separated EPS was dialyzed (3.5 kDa MWCO; SnakeSkin Dialysis Tubing, NJ, USA) for 48h against ultrapure water, which was changed every 12h. After confirming the presence of protein by spectrophotometry (NanoDrop spectrophotometer, Thermo Fisher Scientific, MA, USA), the completely purified EPS was lyophilized, weighed on a scale to determine the yield, and stored in a freezer at −80°C for further experiments.

### Antioxidant activity

#### ABTS radical scavenging activity

The ABTS^*+^ scavenging capacity of EPS produced by strain L3^T^ was determined according to a previously reported method with some modifications (Yang et al., 2021). Briefly, 7 mM ABTS (2,2′-azino-di-(3-ethylbenzothiazoline-6-sulfonic acid) and 2.45 mM potassium persulfate were mixed at a ratio of 1:1 (v/v), then incubated in the dark for 16h at 25°C to generate ABTS radicals. 0.1 mL each of 1 mg/mL EPS solution and ABTS^*+^ solution was added, incubated for 5 min in a dark room at 37°C, and then measured at 734 nm using a spectrophotometer. Distilled water was used as a blank, and ascorbic acid (PC) was used as a positive control.

#### Hydroxyl radical scavenging activity

The OH^*^ scavenging activity of strain L3^T^ was investigated (Tarannum et al., 2023). Briefly, 40 μL of 9 mM FeSO_4_, 40 μL of H_2_O_2_ (0.03%, v/v), and 20 μL of 9 mM salicylic acid−ethanol solution were mixed. Then, 160 μL of the supernatant of strain L3^T^ was added to the mixture, mixed vigorously, and incubated at 37°C for 30 min. Afterward, absorbance was measured at 510 nm using a spectrophotometer. Distilled water and PC were used as negative and positive controls, respectively, and all experiments were repeated three times. The scavenging of OH^*^ by the EPS from strain L3^T^ was calculated according to the following equation:

Hydroxyl radical scavenging capacity of EPS (%) = [1 - (*A*_1_/*A*_2_)] × 100

where *A*_1_ is the absorbance of the sample, and *A*_2_ is the absorbance of the control.

#### DPPH radical scavenging activity

The DPPH radical scavenging activity of EPS was determined by slightly modifying the method proposed by Kim et al (Kim et al., 2023b). Briefly, samples were dissolved in distilled water at concentration of 1 mg/mL. After adding the same volume of 0.2 mM DPPH solution as each sample, it was mixed and incubated for 30 min at 25°C in the dark. Then, absorbance was measured at 517 nm using a spectrophotometer. PC served as a positive control, and distilled water was used as a negative control. The scavenging of DPPH radicals by the EPS from strain L3^T^ was calculated according to the following equation:

DPPH radical scavenging capacity of EPS (%) = [1 − (*A*_1_ − *A*_2_)/*A*_3_] × 100

where *A*_1_ is the absorbance of the DPPH solution mixed with the EPS solution, *A*_2_ is the absorbance of the DPPH solution, and *A*_3_ is the absorbance of the control.

### Statistical analysis

All statistical analyses were performed using GraphPad Prism software version 11.0 for Windows (GraphPad Software, Inc., San Diego, CA, USA). According to Duncan’s test, significant differences between treatment groups were: *P < 0.05, **P < 0.01, ***P < 0.001.

## Results

### Isolation and identification of strains

**Figure 1:**
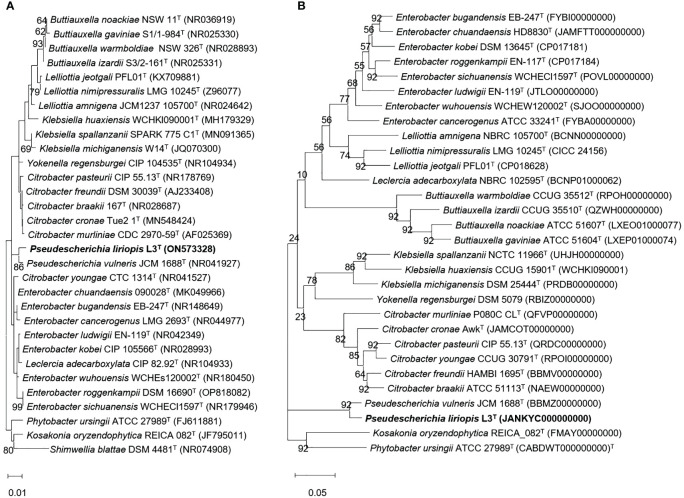
**(A)** Neighbor-joining (NJ) phylogenetic tree of strain L3^T^ within the genus *Pseudescherichia*. Bootstrap values are shown as percentages of 1,000 replicates (only values >50% are shown). Filled circles indicate that the corresponding nodes were recovered in trees generated with the maximum-likelihood (ML), and maximum-parsimony (MP) algorithms. Empty circles indicate that the corresponding nodes were recovered using the ML algorithm. Bar, 0.02 substitutions per nucleotide position. **(B)** Phylogenomic tree was reconstructed using the coding sequences of 92 protein clusters showing the position of strain L3^T^ among closely related species. Parentheses indicate the NCBI accession number for the genome of each strain.

About 11 microbial colonies were obtained from fermented fruit samples. 16S rRNA gene sequencing and morphological analysis were performed. Additionally, we were able to select six types of bacteria presumed to secrete EPS around cells. These six strains were tested on media with different concentrations of NaCl and polyethylene glycol (PEG) to investigate whether they would grow under salinity and drought stress conditions (**Supplementary Table S1**) (Kim et al., 2023c, 2023a). Through repeated experiments, strain L3^T^ was shown to maintain sustainable growth in salinity conditions up to 1.198 M NaCl. It was also selected for PGP testing because it maintained consistent growth even under 22% PEG conditions. Molecular identification of strain L3^T^ was performed by analyzing the 16S rRNA gene base sequence and whole genome sequence of strain L3^T^. Strain L3^T^ showed the highest 16S rRNA gene similarity to *Buttiauxella izardii* CCUG 35510^T^ strain at 98.63%, followed by 98.56% and 98.56% similarity with the *Enterobacter ludwigii* EN-119^T^ and *Pseudescherichia vulneris* NBRC 102420^T^ strains, respectively. The phylogenetic tree was reconstructed using the 16S rRNA gene sequence. Strain L3^T^ showed clustering with *P. vulneris* NBRC 102420^T^. In addition, the phylogenetic tree also showed that it was clustered with *P. vulneris* NBRC 102420^T^. These results indicated that strain L3^T^ was a novel species of the genus *Pseudescherichia* (**Figure 1A**). The bootstrap values shown in the phylogenetic tree and phylogenomic tree were 86 and 92, respectively, which support the above results (**Figures 1A, B**). Therefore, to place strain L3^T^ in the phylogenetically correct position, we performed a taxonomic comparison with the closest species, *P*. *vulneris* NBRC 102420^T^ (**Supplementary Table S2**). Although these two *Pseudescherichia* species showed mostly similar experimental results, only strain L3^T^ was found to grow at pH 4.0, 7% NaCl, and 15°C, revealing the differences between the two strains. In addition, according to the results of the API 20NE test, the only other difference was whether _L_-arginine was hydrolyzed. The almost full-length 16S rRNA gene sequence (1470 bp) of strain L3^T^ was deposited in the GenBank database under the accession number ON573328 and the whole genome sequence JANKYC00000000, respectively.

### Genomic features of strain L3^T^

The draft genome sequence of strain L3^T^ contained 10 contigs, with a total size of 4,304,575 bp, and encoded 4,131 genes in total, 3,989 of which were protein-coding genes. The genome information of strain L3^T^ and its related species is detailed in **Supplementary Table S1**. CheckM version 1.2.2 revealed that the completeness of the strain L3^T^ genome was 100.0%, with a contamination level of 0%. After calculating average nucleotide identity (ANI) values with FastANI using orthogonal mapping, we visualized the genome conservation of two strains that are phylogenetically close to strain L3^T^ (**Supplementary Figure S1**). The results showed the most orthologous mapping between strain L3^T^ and *P*. *vulneris*, suggesting that they are the closest species, and there were relatively few orthologous mappings between strain L3^T^ and *B*. *izardii* and *E*. *ludwigii*, respectively. As shown in **Supplementary Table S3**, ANI and digital DNA–DNA hybridization (dDDH) comparisons between strain L3^T^ and 29 genomes were performed. The ANI and dDDH values between strain L3^T^ and the *P*. *vulneris* genome were 55.1% and 93.9%, those between strain L3^T^ and the *B*. *izardii* genome were 19.9% and 75.9%, and those between strain L3^T^ and the *E*. *ludwigii* genome were 22.4% and 79.5%, respectively. The recommended cutoff values for delineating novel species are 70% (dDDH) and 95% (ANI). The Antibiotic and Secondary Metabolite Analysis Shell (antiSMASH) server revealed a secondary metabolite biosynthetic gene cluster for non-ribosomal peptide metallophores, non-ribosomal peptide synthetase, two other unspecified ribosomally synthesized and post-translationally modified peptides, redox-cofactors, such as pyrroloquinoline quinone (PQQ), and terpene. A comparison of secondary metabolite predictions between strain L3^T^ and phylogenetically close species can be seen in **Supplementary Figure S1B**.

### Plant growth promotion characterizations

**Figure 2:**
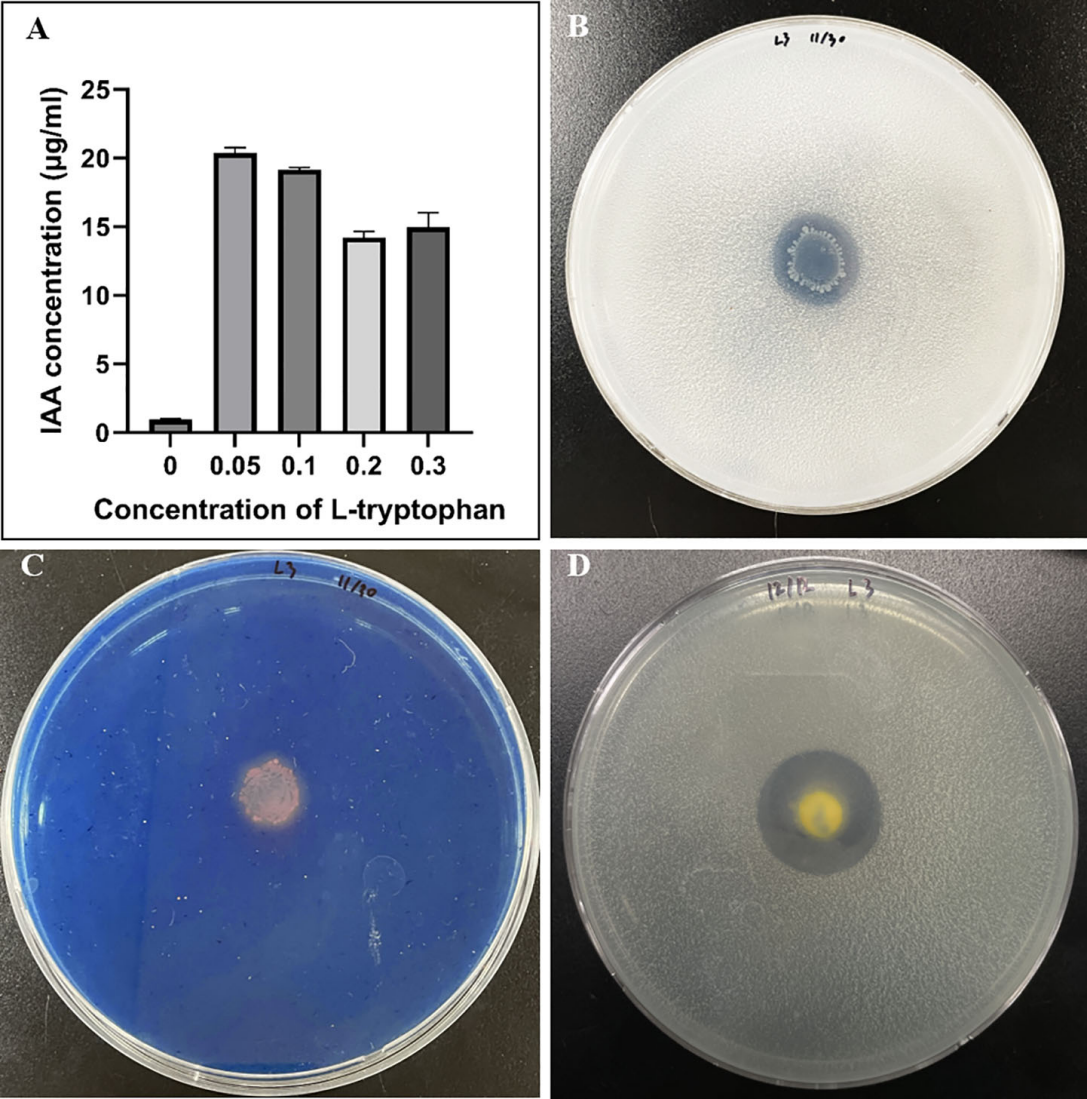
Traits associated with plant growth promotion ability of strain L3^T^. **(A)** IAA production is dependent on the concentration of tryptophan. **(B)** Phosphate solubilization assay (PVK agar plate). **(C)** Siderophore qualitative assay. **(D)** Nitrogen fixation.

Strain L3^T^ produces IAA and has been shown to perform siderophore production, phosphate solubilization, and nitrogen fixation. Confirmation of IAA production was measured using a slight modification of the method described by Gordon and Weber (Gordon and Weber, 1951). The IAA value was highest at 20.37 ± 0.96 μg/mL when 0.05% _L_-tryptophan was included. When the _L_-tryptophan concentration was 0.1%, 0.2%, and 0.3%, the IAA concentration values were 19.16± 0.14, 14.22 ± 0.38, and 14.97 ± 0.91 μg/mL, respectively, confirming that the IAA concentration did not increase as the _L_-tryptophan concentration was increased (**Figure 2A**). Strain L3^T^ had the ability to secrete IAA, but only synthesized IAA in the presence of _L_-tryptophan and could not produce it alone. Strain L3^T^ showed clear areas around colonies grown on Pikovskaya (PVK) agar plates, indicating its ability to utilize inorganic phosphate in the medium (**Figure 2B**). Additionally, the L3^T^ strain grows quickly on PVK agar plates and produces halozone quickly within 24h. The development of a halo around the bacterial colony of strain L3^T^ grown on chrome azurol S (CAS) plate medium indicated its ability to produce siderophores (**Figure 2C**). Strain L3^T^ was able to grow in 1 day on a nitrogen medium at 30°C, indicating its nitrogen fixation ability (**Figure 2D**).

### Effect of strain L3^T^ on the growth of plant

**Figure 3:**
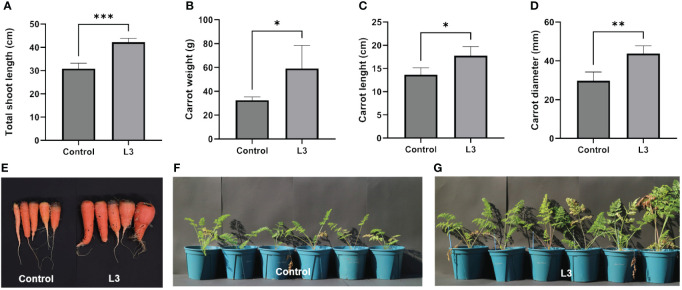
Effect of inoculation with strain L3^T^ on growth promotion of carrot plants. **(A)** Total shoot length of carrot plants in L3^T^-inoculated and control groups. **(B)** Carrot weight of L3^T^-inoculated group and control group. **(C)** Effect of carrot length in L3^T^-inoculated group and control group. **(D)** Effect of carrot diameter according to strain L3^T^ inoculation, **(E)** carrots with leaves and soil removed, **(F)** control group not inoculated with L3^T^, **(G)** group inoculated with L3^T^. Data are presented as mean + SD of five replicates. *P < 0.05, **P < 0.01, ***P < 0.001.

Based on IAA production, siderophore production, phosphate solubilization, and a nitrogen fixation study, it was investigated whether strain L3^T^ could promote carrot (*D. carota* subsp. *sativus*) plant growth. The results confirmed that the stem length, carrot diameter, carrot weight, and carrot length were significantly increased (**Figures 3A–D**). Inoculation with strain L3^T^ increased the weight of carrots by 81.5% compared to the control group, and the shoot length, diameter, and length of carrots increased by 30.13%, 47.06%, and 36.98%, respectively. These results suggest that strain L3^T^ promoted the growth of carrots. In addition, among the results of the pot experiment, the PGP ability of strain L3^T^ was confirmed in photos of the inoculated carrots with leaves and soil removed (**Figure 3E**). There were also clear differences in leaf length and abundance between the L3^T^-inoculated group and the control group (**Figures 3F, G**).

### Effect of strain L3^T^ inoculation on alleviating salinity and drought stress

**Figure 4:**
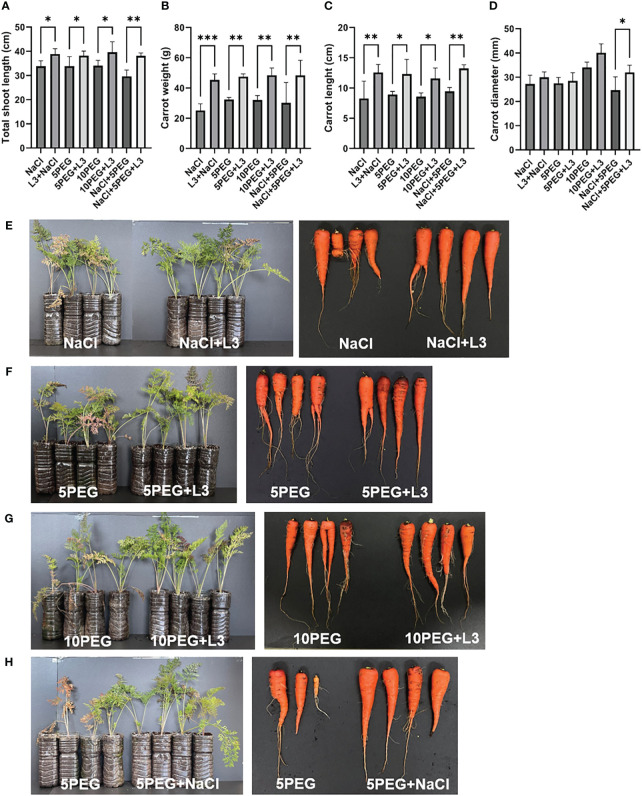
Inoculation effect of strain L3^T^ under drought and NaCl stress: **(A)** effect of total shoot length under drought and salt stress, **(B)** carrot weight, **(C)** carrot length, and **(D)** carrot diameter. Data are presented as mean + SD of four replicates. Effect of inoculation of strain L3^T^ on growth promotion of carrot plants under salinity and drought stress. **(E)** NaCl (0.513 M), **(F)** 5% PEG, **(G)** 10% PEG, **(H)** 5% PEG + NaCl (0.513 M). *P < 0.05, **P < 0.01, ***P < 0.001.

The plants inoculated with strain L3^T^ cultures showed an improvement in plant growth under salinity and drought stress. When inoculated with the L3^T^ strain under NaCl stress (0.514 M), carrots’ total shoot length increased by 14.84% compared to the control group (**Figure 4A**). Moreover, unlike the plants inoculated with strain L3^T^, the leaves of the control plants turned yellow due to stress caused by salt (**Figure 4E**). Drought stress was assessed by treatment with PEG at 5% and 10% concentrations (referred to as the 5PEG and 10PEG groups, respectively). Plants inoculated with strain L3^T^ under drought stress showed a 12.84% (5% PEG) and 16.37% (10% PEG) increase in carrots’ total shoot length growth compared to the control group (**Figure 4A**). In addition, carrots’ weight increased by 80.01%, 46.52%, and 50.98% under salinity, 5% PEG, and 10% PEG conditions, respectively (**Figure 4B**). The 5PEG group also had yellow and dry leaves compared to the L3^T^-inoculated group (**Figure 4F**). Moreover, in the 10PEG group, the total length of shoots was significantly reduced, leaves dried, and stems also dried and tilted to the side (**Figure 4G**). Carrot length also increased by 51.96% (0.514 M NaCl), 38.1% (5% PEG), and 35.28% (10% PEG), under the indicated stress compared to the control group (**Figure 4C**). Furthermore, there was an increase in the diameter of the thickest part of carrots compared to the control group, albeit the value was not significant (except for the NaCl + 5PEG group; **Figure 4D**). Inoculation with strain L3^T^ under conditions of overlapping salt and drought stress significantly increased total plant sprout length, carrot length, carrot weight, and carrot diameter by 28.54%, 40.21%, 60.49%, and 29.73%, respectively, compared to the control group (**Figures 4A–D**). In contrast, in the control group, one of the plants did not grow, and the overall length of the leaves and the size of the edible parts of the carrots also showed differences from the L3^T^-inoculated group (**Figure 4H**). These results suggest that inoculation with strain L3^T^ may be a countermeasure against crop damage caused by salinity and drought.

### Genome analysis and plant growth promotion insight

As a result of this study, the new strain L3^T^ promoted the growth of carrots. The genome of strain L3^T^ was examined to identify genes involved in plant growth promotion. A variety of PGPB exhibit IAA production, phosphate solubilization, nitrogen fixation, and siderophore production. In the strain L3^T^ genome, a set of genes for IAA production was discovered, and IAA production was confirmed experimentally, showing consistency between these two results (**Table 1**). A set of genes responsible for phosphate solubilization and transport were also included in the genome (**Table 1**). Additionally, it was experimentally confirmed that the L3^T^ strain dissolves insoluble phosphate on PVK agar plates. Bacteria that solubilize phosphate release gluconic acid, which converts the insoluble phosphate into a soluble form (Ahemad and Kibret, 2014). Additionally, PQQ is known to be a PGP factor that is associated with antioxidant properties (Choi et al., 2008). The set of PQQ-related genes was contained in the strain L3^T^ genome (**Table 2**). In the draft genome of strain L3^T^, genes related to nitrogen metabolism, nitrogen fixation, and siderophore production were found, of which only one gene was related to nitrogen fixation (**Table 2**).

**Table 1:** Genes involved in IAA production and phosphate solubilization predicted from the strain L3^T^ genome.

Properties	Name	Locus tag
**Indole-3-acetic acid**	Bifunctional anthranilate synthase glutamate amidotransferase component *TrpG*, anthranilate phosphoribosyltransferase *TrpD*	NR795_07870
	Anthranilate synthase component 1	NR795_07865
	Bifunctional indole-3-glycerol-phosphate synthase *TrpC*, phosphoribosyl anthranilate isomerase *TrpF*	NR795_07875
	N-methyl-L-tryptophan oxidase *solA*	NR795_03815
	Tryptophan synthase subunit beta *trpB*	NR795_07880
	Tryptophan synthase subunit alpha *trpA*	NR795_07885
	Tryptophan–tRNA ligase *trpS*	NR795_15650
	Tryptophan permease *mtr*	NR795_18040
**Phosphate solubilization**	Phosphate response regulator transcription factor *PhoB*	NR795_00415
	Phosphate regulon sensor histidine kinase *PhoR*	NR795_00420
	Phosphate starvation-inducible protein *PhoH*	NR795_03675
	Phosphate signaling complex protein *PhoU*	NR795_17295
	Phosphate ABC transporter ATP-binding protein *PstB*	NR795_17300
	Phosphate ABC transporter permease *PstA*	NR795_17305
	Phosphate ABC transporter permease *PstC*	NR795_17310
	Phosphate ABC transporter substrate-binding protein *PstS*	NR795_17315
	Octa prenyl diphosphate synthase *ispB*	NR795_17835
	Phosphonate C-P lyase system protein *PhnH*	NR795_14870
	Exopolyphosphatase *ppx*	NR795_11090
	Polyphosphate kinase 1 *ppk1*	NR795_11085
	Phosphate acetyltransferase *pta*	NR795_10460
	Glucose-6-phosphate dehydrogenase *zwf*	NR795_08600

**Table 2:** Genes involved in pyrroloquinoline quinone biosynthesis, nitrogen fixation, nitrogen metabolism, and siderophore production predicted from strain L3^T^.

Properties	Name	Locus tag
**Pyrroloquinoline quinone**	Pyrroloquinoline quinone precursor peptide *PqqA*	NR795_11860
	Pyrroloquinoline quinone biosynthesis protein *PqqB*	NR795_11865
	Pyrroloquinoline-quinone synthase *PqqC*	NR795_11870
	Pyrroloquinoline quinone biosynthesis peptide chaperone *PqqD*	NR795_11875
	Pyrroloquinoline quinone biosynthesis protein *PqqE*	NR795_11880
	Pyrroloquinoline quinone biosynthesis protein *PqqF*	NR795_11885
**Nitrogen fixation**	Pyruvate:ferredoxin (flavodoxin) oxidoreductase *NifJ*	NR795_06880
**Nitrogen metabolism**	P-II family nitrogen regulator *glnK*	NR795_00695
	Nitrogen assimilation transcriptional regulator *NAC*	NR795_09305
	Nitrogen regulatory protein P-II *glnB*	NR795_11375
	PTS IIA-like nitrogen regulatory protein PtsN	NR795_17750
	Nitrogen regulation protein NR(I) *glnG*	NR795_19485
	Nitrogen regulation protein NR(II) *glnL*	NR795_19490
**Siderophore production**	TonB-dependent siderophore receptor	NR795_00925
	TonB-dependent siderophore receptor	NR795_01210
	TonB-dependent siderophore receptor	NR795_01490
	Fe(3+)-siderophore ABC transporter permease *fepD*	NR795_01525
	TonB-dependent siderophore receptor	NR795_08280
	Catecholate siderophore receptor *cirA*	NR795_10045
	Siderophore-iron reductase *fhuF*	NR795_13900
	Siderophore-interacting protein	NR795_18295

### ABTS, hydroxyl, and DPPH radical scavenging by EPS

**Figure 5:**
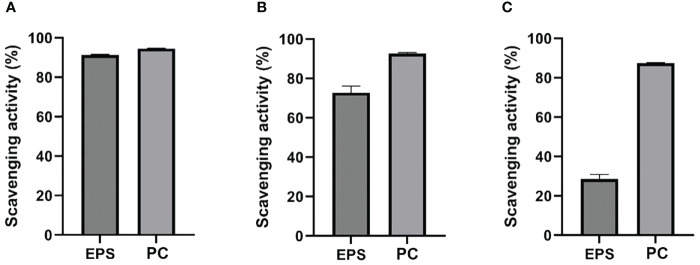
Comparison of the scavenging effects of EPS from *Pseudescherichia liriopis* L3^T^: **(A)** ABTS^*+^, **(B)** OH^*^, and **(C)** DPPH^*^ with that of ascorbic acid (PC).

The antioxidant potential of partially purified EPS was tested. The ABTS^*+^, OH^*^, and DPPH^*^, scavenging activities were investigated *in vitro* and compared with the PC. At a concentration of 1 mg/mL, EPS, and PC had almost similar ABTS^*+^ scavenging activities (91.2% and 94.6%; **Figure 5**), and the results indicated that EPS produced by strain L3^T^ has excellent scavenging activity toward ABTS^*+^. The OH^*^ scavenging activity of EPS (72.8%) was lower than that shown by PC (92.6%) but was still excellent (**Figure 5**). As shown in **Figure 5** at concentrations of 1 mg/mL, PC showed high DPPH^*^ scavenging ability (87.5%) and EPS showed less (28.5%).

### Composition and monosaccharide analysis of EPS

**Figure 6:**
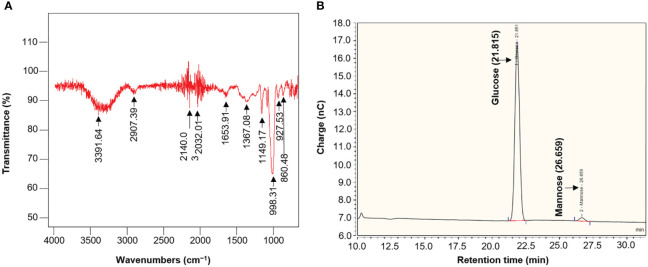
**(A)** ATR-FTIR spectra of EPS from *Pseudescherichia liriopis* L3^T^. **(B)** Monosaccharide composition of EPS from *P*. *liriopis* L3^T^ by UHPLC analysis.

ATR-FTIR spectra of EPS produced from strain L3^T^ were collected using a Smiths 70v spectrometer in the spectral range of 650–4000 cm^−1^. This allowed us to characterize the covalent bond information and confirm the presence of functional groups (**Figure 6A**). A strong absorption band was observed near 3391 cm^−1^ in the FTIR spectrum, which is due to the ─OH group expansion vibration and indicates that the polymer is EPS (Wang et al., 2018). The absorption band appearing at 2937.59 cm^−1^ typically represents the stretching vibration of a hexose, such as glucose, or a methylene group (C─H) (Rajoka et al., 2022). The absorption bands around 1653 and 1367 cm^−1^ are attributed to the stretching vibration of the C═O bond and symmetric CH_3_ bending, respectively (Freitas et al., 2009). A peak presumed to be COO^−^ vibration, evidence of sulfuric acid ester, appeared around 1367 cm^−1^ (Gupta et al., 2021). The absorption band representing the stretching vibration of C─O and the angle change vibration of O─H, indicating pyranose-type glucose and carbohydrates, was observed at approximately 1149 cm^−1^ (Bremer and Geesey, 2009). The absorption bands in the region 900–1150 cm^−1^ are characteristic of carbohydrates and are attributed to C─O─C and C─O stretching bending vibrations (Na et al., 2010). The absorption peak around 860 cm^−1^ is largely formed by α-glycosidic bonds (Cao et al., 2020). As shown in the chromatogram in **Figure 6B**, the peaks obtained by UHPLC represent the monosaccharide profile of EPS hydrolyzed with 2 M trifluoroacetic acid. Two peaks corresponding to glucose (21.58 min) and mannose (26.66 min) were observed in acid-hydrolyzed EPS, indicating a relative molar ratio of 33.6:1.0.

### Thermogravimetric analysis

**Figure 7:**
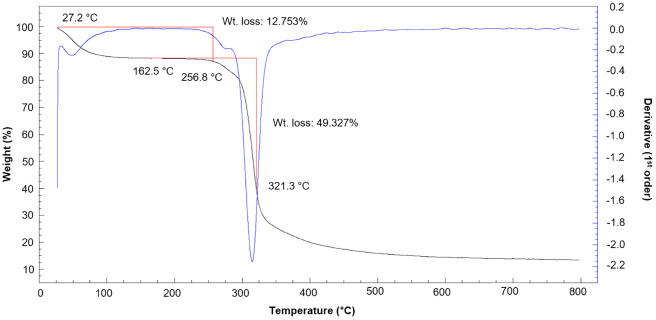
Thermogravimetric analysis (TGA) of the EPS produced by *Pseudescherichia liriopis* L3^T^. The black line represents TGA, and the blue line represents differential thermal analysis.

The thermal properties of EPS are important for its commercial use. In this study, Thermogravimetric (TGA) of EPS produced by the novel strain L3^T^ was performed from 25°C to 800°C (**Figure 7**). The first decrease occurred at 27.2°C, which was a mass loss due to gelatinization and swelling, mainly associated with water loss (Fagerson, 1969). Afterward, a loss of 12.75% of the total weight of EPS was confirmed up to 256.8°C, and the greatest energy release occurred between 300 and 321.3°C. The subsequent thermal weight loss of EPS represents a mass loss of approximately 49.33% at 321.3°C.

## Discussion

Our results of 16S rRNA gene sequence-based phylogenetic analysis, genome comparison of strain L3^T^, and the physiological characteristics of strain L3^T^ showed that it represents a novel species in the genus *Pseudescherichia*. Strain L3^T^ showed 98.63%, 98.56%, and 98.56% 16S rRNA gene sequence similarity to *B. izardii* CCUG 35510^T^, *E. ludwigii* EN-119^T^, and *P. vulneris* NBRC 102420^T^, respectively. The ANI values between novel strain L3^T^ and *B. izardii* CCUG 35510^T^, *E. ludwigii* EN-119^T^, and *P*. *vulneris* NBRC 102420^T^ were 75.9%, 79.5%, and 93.9%, respectively, with respective dDDH values of 19.9%, 22.4%, and 55.1%. The ANI values were lower than the ANI threshold of 95%, strongly supporting strain L3^T^ as a novel species (Chun et al., 2018).

The main goal of this research work is to find ba that promote plant growth, as well as those that have both NaCl and drought stress tolerance. A total of six strains were identified, and strain L3^T^, which produced EPS and grew at a concentration of 20% PEG and 6% NaCl, was designated. This novel strain was able to perform phosphate solubilization, IAA production, siderophore synthesis, and nitrogen fixation. It also had the ability to secrete IAA but only synthesized IAA in the presence of _L_-tryptophan and could not produce it alone. In addition, IAA synthesis does not increase as _L_-tryptophan concentration increases but rather is synthesized in the largest amount when 0.5% _L_-tryptophan is present, indicating that it is not concentration dependent. *Enterobacter cloacae* MG00145, another genus in the same family, shows an IAA production of approximately 17.7 μg/mL in the presence of tryptophan. In comparison, strain L3^T^ shows a higher value (20.37 ± 0.96 μg/mL) (Panigrahi et al., 2020). Auxin is a major plant hormone that plays an important role in regulating plant growth, including leaves, fruits, flowers, and germination (Bottini et al., 2004; Hayat et al., 2010). Auxin production by our strain L3^T^ was consistent with PGP ability in several species (Chhetri et al., 2022; Tsavkelova et al., 2024). The ability of phosphate solubilization is crucial as it enhances the availability and absorption of essential mineral nutrients by plants (Arif et al., 2017). Genera known as siderophore producing bacteria, such as *Pseudomonas*, *Bacillus*, and *Rhizobium*, improve iron absorption, making plants stronger and more effective in suppressing pathogens (Abo-Zaid et al., 2020; Sun et al., 2022; Xie et al., 2024). We experimentally demonstrated the ability of the L3^T^ strain to synthesize siderophores, fix nitrogen, solubilize phosphate, and synthesize IAA and identified IAA production, phosphate solubilization, nitrogen fixation, and siderophore gene clusters in the NCBI annotation and genes associated with plant growth promotion. It is suggested that the collective effects of siderophore production, nitrogen fixation, and phosphate solubility may contribute to the growth enhancement shown by inoculation of the novel species L3^T^ in carrot plants. Therefore, the two results appear to be consistent.

Additionally, various clusters related to PQQ biosynthesis were discovered. PQQ is known to have antioxidant properties and scavenge reactive oxygen species, promoting plant growth and phosphate solubilization (Choi et al., 2008). However, although PQQ-related clusters were predicted in the genome, additional experiments are needed to determine the mechanism(s) by which strain L3^T^ affects PQQ production and plant growth. Based on these results, the ability of the strain to promote plant growth was verified by inoculating strain L3^T^ into carrot plants. When carrot plants were inoculated with strain L3^T^, the carrot plants’ weight, length, diameter, and leaf length increased significantly. The key among these was that the edible part of the carrots increased as the length, weight, and diameter increased. In addition, strain L3^T^ affected leaf length, weight, and length of carrots even under salt and drought stress conditions, respectively. The size, length, diameter, and leaf length of carrot plants increased compared to control plants subjected to salt and drought stress. Plants inoculated with strain L3^T^ showed a clear increase in leaf length, leaf weight, carrot length, and carrot thickness compared to the control group, even under conditions where salt and drought stress occurred simultaneously.

Crop yield and growth are being hindered due to soil salinization, and climate change conducive to salt accumulation might increase the proportion of salinized land (Ilangumaran and Smith, 2017). Agricultural lands with high salt content have a low phosphorus content. Therefore, it is essential to use fertilizers that contain a large amount of phosphorus, but phosphorus can cause water pollution and accumulation of toxic elements during long-term use (Alori et al., 2017). PGPB live in the rhizosphere and internal tissues of plants and promote crop growth and yield through various PGP mechanisms (Pii et al., 2015). This is an environmentally friendly strategy as it leads to a reduction in soil and water pollution resulting from the use of chemical and nitrogen fertilizers (Aminun Naher et al., 2015). Instead of fertilizer, using PGPB, such as strain L3^T^, dissolves soil phosphate, providing a sustainable alternative to promote crop growth and increase yields. Drought is a plant stress that inhibits crop growth and has a negative impact on yield (Zhu, 2016). Therefore, the study of PGPB to improve drought stress is important as plant stress directly affects agricultural production.

Strain L3^T^ also grew well in R2A broth containing 20% PEG. This showed potential for improving plant resistance to drought stress. We demonstrated that inoculation of carrot plants with the L3^T^ strain under drought stress promoted plant growth compared to the control group that was not inoculated with the strain. This indicates that the L3^T^ strain has the potential to positively affect water retention in rhizosphere soils, which may be related to the EPS produced by strain L3^T^. EPS produced by bacteria may be involved in slowing the rate of dehydration of plants by retaining moisture, which should be studied further. Strain L3^T^, an EPS-producing bacterium, not only suggests that it may be useful in the development of biological inoculants to improve abiotic stress in plants but also shows strong antioxidant ability, making it worthy of application in various industries. Microbial EPS production is a physiological adaptation that allows survival under stressful conditions. Therefore, the ability of bacterial cells to produce EPS is associated with the drought resistance of bacteria (Sandhya et al., 2009). The production of EPS on PGP strains plays a crucial role in managing salt stress and can be an effective solution. EPS from chelates with ionic metals such as Na^+^ and Cl^-^, binding directly to positive ions like Na^+^. This action stabilizes the ions and mitigates NaCl stress in plants (Kumar et al., 2021). To the best of our knowledge, this is the first study of PGP activity among *Pseudescherichia* species. So far, many PGP studies have been conducted on improving NaCl stress and drought stress (Kumar et al., 2021; Ashry et al., 2022). However, even when both stresses were applied simultaneously, our strain L3^T^ showed the ability to promote plant growth. When inoculated with the strain L3^T^ under conditions where salinity and drought stress overlapped, the total sprout length, carrot length, carrot weight, and carrot diameter of the plants were confirmed to increase by 28.54%, 40.21%, 60.49%, and 29.73%, respectively, compared to the control group.

We also investigated the characteristics of EPS produced by the PGPB strain L3^T^. AIR-FTIR analysis was performed to determine the presence or absence of functional groups in EPS. EPS exhibited peaks typical of polysaccharides, suggesting that this polymer is EPS (Wang et al., 2010). It was confirmed that the EPS from strain L3^T^ consists mainly of glucose and some mannose, which may be heteropolysaccharides. It is similar to EPSe5 produced by lactic acid bacteria but has a slightly different monosaccharide composition (Liu et al., 2023). Additionally, the monosaccharide composition of EPS produced by strain L3^T^ suggests that it is different from EPS produced by other strains (Kim et al., 2022; Liu et al., 2023). EPS produced by microorganisms is regulated by several genetic factors, carbon sources, and environmental variables and may differ in composition from EPS produced by other strains, even of the same species (Pachekrepapol et al., 2017). Recent plant-related studies have focused on the interactions between plant and microbial secondary metabolites.

## Conclusion

PGPB studies are important from environmental and agricultural perspectives. In this study, the new strain L3^T^ was used to demonstrate its ability to promote plant growth in carrot plants. As a result, the length, weight, and leaf length of the carrots increased, increasing the edible portion. In addition, inoculation with strain L3^T^ was confirmed to improve these problems associated with drought and salt stress in plants. In addition, biotechnologically important EPS was extracted and purified from *P. liriopis* sp. nov. L3^T^. EPS produced by bacteria obtained from fermented *L. platyphylla* fruit was also studied using FTIR, TGA, and UHPLC analysis. The EPS we studied showed excellent antioxidant properties, and its thermal stability was emphasized, supporting its use in multiple industrial applications. The practical application of this research is important for both agriculture and industry. Integrating the L3T strain into agricultural practices can improve crop yields, especially in areas vulnerable to drought and salt stress. This can lead to more sustainable and resilient agricultural systems. Sustainable agriculture: using natural plant growth promoters like L3^T^ can promote more environmentally friendly agricultural practices by reducing reliance on chemical fertilizers and pesticides. Although the results of this study are promising, several potential limitations need to be addressed. Further research is needed to understand the structure of EPS, its biological activity, and its correlation with plant growth. Additional studies are needed to understand the detailed correlation with plant growth promotion, structure, and biological activity of EPS. In future studies, we should investigate the mechanisms by which EPS promotes plant growth and enhances abiotic stress at the cellular and molecular levels. Because agricultural environments have different conditions depending on the region, plant growth tests must be conducted in a wide range of fields, and microbial community dynamics and nutrient cycling must also be studied to determine how they affect the soil. Economic aspects are also important and the financial efficiency of using L3^T^ strains in agriculture must be evaluated.

## Data availability statement

The datasets presented in this study can be found in online repositories. The names of the repository/repositories and accession number(s) can be found in the article/**Supplementary Material**.

## Author contributions

IK: Conceptualization, Data curation, Formal analysis, Investigation, Validation, Writing – original draft. HW: Data curation, Investigation, Methodology, Writing – review & editing. GC: Data curation, Methodology, Writing – review & editing. SP: Data curation, Writing – review & editing. TS: Writing – review & editing, Funding acquisition, Resources, Supervision.
