# Supplementary material for: A novel exopolysaccharide-producing bacterium, Pseudescherichia liriopis sp. nov. isolated from Liriope platyphylla, enhances the growth of Daucus carota subsp. sativus under drought and salinity stress
Source: Front Plant Sci. 2024 Jul 16;15:1417639. doi: 10.3389/fpls.2024.1417639 (PMC11286387; doi:10.3389/fpls.2024.1417639)
Supplement: Supplementary file 1 [file DataSheet_1.docx]

Supplementary Material

**A novel exopolysaccharide-producing bacterium, *Pseudescherichia liriopis* sp. nov. isolated from *Liriope platyphylla*, enhances the growth of *Daucus carota* subsp. *sativus* under drought and salinity stress**

**Inhyup Kim^1^, Haejin Woo^1^, Geeta Chhetri^1^, Sunho Park^1^, and Taegun Seo^1,*^**

^1^Department of Life Science, Dongguk University-Seoul, Goyang 10326, Republic of Korea

*** Correspondence:**

Corresponding Author: Taegun Seo

E-mail: [tseo@dongguk.edu](mailto:tseo@dongguk.edu)

Tel.: +82-31-961-5135


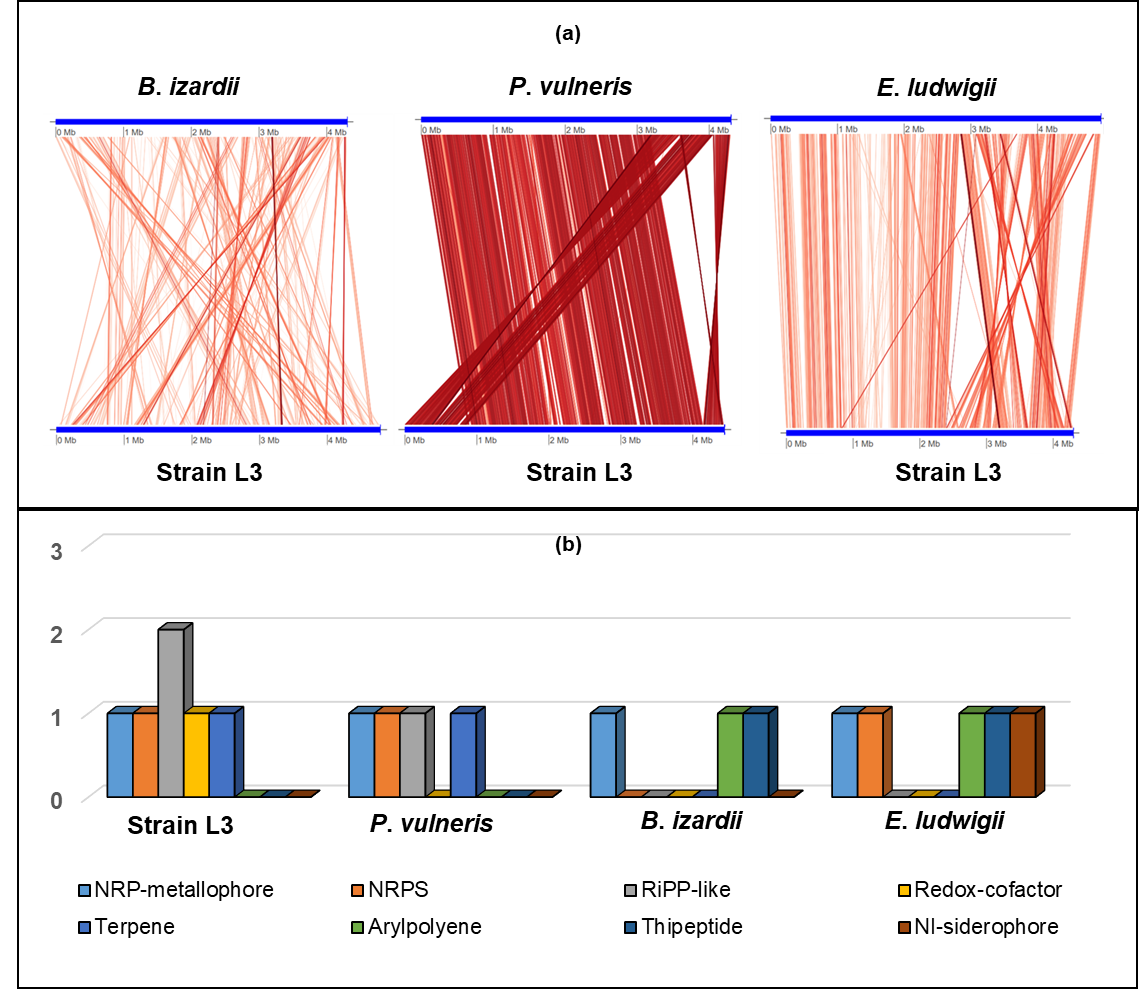


**Fig. S1.** (a) Illustration representing FastANI's workflow between the novel strain L3^T^ genome and a phylogenetically close reference genome. (b) The results of antiSMASH show secondary metabolites of strain L3^T^ and phylogenetically related species.

| **Bacterial strains** | **20%PEG** | **6% NaCl** | **EPS producing** |
| --- | --- | --- | --- |
| RP10 | – | – | + |
| RP14 | – | – | + |
| L1 | – | + | + |
| L3 | + | + | + |
| L8 | + | – | + |
| L10 | – | + | + |

**Table S1**. Qualitative analysis of six isolates for EPS production, PEG resistance, and NaCl resistance. RP10 = *Sphingomonas liriopis* RP10^T^, RP14 = *Mesorhizobium liriopis* RP14^T^, L1 = Rhizobium sp., L3 = *Pseudescherichia liriopis* L3^T^, L8 = *Lactilactobacillus sakei* subsp. *sakei* JCM 1157^T^, L10 = *Phyllobacterium* RBR8, + =positive, and – = negative.

| **Characterization** | **1** | **2** |
| --- | --- | --- |
| Range of growth |  |  |
| Temperature (°C) | 15–45 | 18–45 |
| NaCl (%, w/v) | 0–7 | 0–6 |
| pH | 4–13 | 5–13 |
| Hydrolysis of |  |  |
| _L–_Arginine | + | – |
| Genome accession number | JANKYC000000000 | UGGL00000000 |
| Genome size (bp) | 4,304,575 | 4,442,322 |
| Contigs | 10 | 2 |
| N50 | 2,389,350 | 4,427,509 |
| Total genes | 4131 | 4360 |
| Protein-coding genes | 3989 | 3809 |
| rRNAs (5S, 16S, 23S) | 5, 2, 2 | 8, 7, 7 |
| ncRNAs | 12 | 12 |
| tRNAs | 72 | 81 |
| Pseudo genes | 49 | 436 |
| DNA G+C content | 56.5* | 56.5* |

**Table S1.** Differentiating characteristics of strain L3^T^ and other *Pseudescherichia* species. 1, L3^T^; 2, *Pseudescherichia vulneris* JCM 1688^T^. All dates are from this research. On the API 20NE strips, all strains were negative for hydrolysis of urea and gelatine and assimilation of caprate, adipate, trisodium citrate, and phenylacetic acid. All strains were positive for hydrolysis of *β*–galactosidase and aesculin; assimilation of _D_–glucose, _D_–mannose, _L_–arabinose, _D_–mannitol, _D_–maltose, *N*-acetyl-glucosamine, potassium gluconate, and malate. *, From whole genome sequencing for genomic sequences; +, Positive; –, Negative.

| **Strains (NCBI genome accession number)** | **dDDH** | **ANI** |
| --- | --- | --- |
| *Buttiauxella izardii* CCUG 35510^T^ (QZWH00000000) | 19.9 | 75.9 |
| *Enterobacter ludwigii* EN-119^T^ (JTLO00000000) | 22.4 | 79.5 |
| *Pseudescherichia vulneris* JCM 1688^T^ | **55.1** | **93.9** |
| *Buttiauxella noackiae* ATCC 51607^T^ (LXEO01000077) | 19.8 | 75.3 |
| *Citrobacter braakii* ATCC 51113^T^ (UBGZ00000000) | 21.8 | 78.3 |
| *Citrobacter pasteurii* CIP 55.13^T^ (QRDC00000000 | 23.1 | 80.3 |
| *Buttiauxella warmboldiae* CCUG 35512^T^ (RPOH00000000) | 20.5 | 76.8 |
| *Enterobacter kobei* DSM 13645^T^ (CP017181) | 23.6 | 79.8 |
| *Enterobacter chuandaensis* HD8830^T^ (JAMFTT000000000) | 22.8 | 80 |
| *Citrobacter youngae* CCUG 30791^T^ (RPOI00000000 | 21.8 | 78.1 |
| *Leclercia adecarboxylata* NBRC 102595^T^ (BCNP01000062) | 22.9 | 79.9 |
| *Klebsiella spallanzanii* NCTC 11966^T^ (UHJH00000000) | 22 | 78.6 |
| *Lelliottia jeotgali* PFL01^T^ (CP018628) | 22.2 | 79.1 |
| *Enterobacter bugandensis* EB-247^T^ (FYBI00000000) | 23 | 79.9 |
| *Enterobacter roggenkampii* EN-117^T^ (CP017184) | 23.1 | 80.3 |
| *Kosakonia oryzendophytica* LMG 26432^T^ (FYBE00000000) | 21.6 | 78.7 |
| *Lelliottia nimipressuralis* LMG 10245^T^ (CICC 24156) | 22.4 | 79.5 |
| *Klebsiella huaxiensis* CCUG 15901^T^ (WCHKI090001) | 22.2 | 78.3 |
| *Citrobacter freundii* HAMBI 1695^T^ (BBMV00000000) | 21.7 | 80.3 |
| *Buttiauxella gaviniae* ATCC 51604^T^ (LXEP01000074) | 19.7 | 75.4 |
| *Lelliottia amnigena* NBRC 105700^T^ (BCNN00000000) | 21.5 | 78.5 |
| *Enterobacter sichuanensis* WCHECl1597^T^ (POVL00000000) | 22.7 | 79.7 |
| *Citrobacter cronae* Awk^T^ (JAMCOT00000000) | 21.8 | 78.4 |
| *Phytobacter ursingii* ATCC 27989^T^ (JAWJAC000000000) | 21.7 | 78.5 |
| *Yokenella regensburgei* DSM 5079^T^ (RBIZ00000000) | 22 | 79 |
| *Enterobacter cancerogenus* ATCC 33241^T^ (FYBA00000000) | 22.8 | 79.8 |

**Table S2.** Phylogenetic tree-based DNA–DNA hybridization (dDDH) and average nucleotide identity (ANI) index for the genomes of 29 species.
